# Comparative evaluation of fracture toughness between zirconia and lithium disilicate ceramics: An *in vitro* study

**DOI:** 10.6026/973206300214796

**Published:** 2025-12-15

**Authors:** N. Simhachalam Reddy, Divya Bekkem, Duggineni Sreenivasulu, Addugala Hemavardhini, J. Lakshmi Sowjanya, B. Chandra Obula Reddy

**Affiliations:** 1 Department of Prosthodontics, G Pulla Reddy Dental College And Hospital, G P R Nagar, Nandyal Road, Kurnool, Andhra Pradesh, India; 2 Department of Prosthodontics, Kamineni Institute of Dental Sciences, Narketpally, Sreepuram, Nalgonda, Telangana, India

**Keywords:** All-ceramic restorations, fracture toughness, lithium disilicate, vickers indentation, zirconia, mechanical properties

## Abstract

Fracture toughness is a critical determinant of long-term success in all-ceramic restorations, yet comparative data between major ceramic systems remain limited. Therefore, it is of interest to evaluate and compare the fracture toughness of yttria-stabilized zirconia and lithium disilicate ceramics. Forty CAD/CAM-fabricated specimens were tested using Vickers indentation and fracture toughness was calculated via the indentation fracture method. Zirconia showed significantly higher fracture toughness (408.61 ± 44.21 MPa.m^1/2^) and hardness than lithium disilicate (359.40 ± 19.00 MPa.m^1/2^) (p <0.0001). Zirconia demonstrates superior crack resistance compared to lithium disilicate, supporting its use in high-stress clinical applications.

## Background:

The evolution of dental ceramics has revolutionized restorative dentistry, with all-ceramic materials increasingly replacing traditional metal-ceramic restorations due to their superior esthetic outcomes, biocompatibility and patient demand for metal-free restorations [1]. Contemporary all-ceramic systems offer optimal light transmission properties, excellent color stability and the ability to mimic natural tooth structure, making them the material of choice for anterior and posterior restorations [2]. However, the clinical success of ceramic restorations depends not only on esthetic performance but critically on their mechanical properties, particularly their ability to withstand functional stresses and resist fracture [3]. Among the various all-ceramic materials available, yttria-stabilized tetragonal zirconia polycrystal (Y-TZP) and lithium disilicate glass-ceramics have emerged as the most widely used systems in contemporary prosthodontic practice [4]. Zirconia has gained prominence due to its exceptional mechanical strength, with flexural strength exceeding 900 MPa, making it suitable for multi-unit posterior fixed dental prostheses [5]. The superior mechanical properties of zirconia are attributed to the transformation toughening mechanism, wherein stress-induced phase transformation from tetragonal to monoclinic crystal structure creates compressive stresses that inhibit crack propagation [6]. Lithium disilicate glass-ceramics, commercially available as IPS e.max system, represent another popular choice for all-ceramic restorations, offering an optimal balance between esthetics and mechanical performance [7]. The material consists of approximately 70% lithium disilicate crystals embedded in a glassy matrix, providing flexural strength of 400-450 MPa and superior translucency compared to zirconia [8]. The microstructure features elongated, needle-like lithium disilicate crystals that contribute to crack deflection and bridging mechanisms, enhancing the material's resistance to fracture [9]. Fracture toughness (K_IC_) represents a critical mechanical property that quantifies a material's resistance to crack propagation and is considered a reliable predictor of clinical performance [10]. Unlike flexural strength, which measures the stress required to initiate failure, fracture toughness evaluates the material's ability to tolerate existing flaws and resist catastrophic failure [11]. Several studies have investigated the mechanical properties of zirconia and lithium disilicate; however, direct comparative evaluations using standardized testing protocols remain limited [12]. Recent systematic reviews have highlighted the need for more comparative data on fracture toughness to guide evidence-based material selection for different clinical scenarios [13]. The Vickers indentation fracture (VIF) method has been widely employed for fracture toughness evaluation of ceramic materials due to its simplicity, cost-effectiveness and requirement of minimal sample preparation [14]. This technique involves creating controlled indentation-induced cracks and measuring their dimensions to calculate fracture toughness values [15]. Despite some limitations regarding absolute accuracy, the VIF method provides reliable comparative data and is particularly useful for ranking materials based on their resistance to crack propagation [16]. Previous investigations have reported fracture toughness values ranging from 2 to 4 MPa.m^1/2^ for lithium disilicate and 4 to 10 MPa.m^1/2^ for Y-TZP ceramics [17]. However, variations in testing methodologies, specimen preparation techniques and commercial brands make direct comparisons challenging [18]. Furthermore, limited data exist comparing specific commercial products commonly used in clinical practice under identical testing conditions. Understanding the comparative fracture toughness of these materials is essential for clinicians to make informed decisions regarding material selection based on clinical indications, loading conditions and patient-specific factors [19]. While zirconia is generally perceived as mechanically superior, quantitative comparative data are necessary to support evidence-based clinical decision-making [20]. Therefore, it is of interest to comparatively evaluate the fracture toughness of yttria-stabilized zirconia and lithium disilicate glass-ceramic using the Vickers indentation method under standardized laboratory conditions.

## Materials and Methods:

## Study design and sample size:

This *in vitro* experimental study was conducted in the Department of Prosthodontics in collaboration with the Materials Testing Laboratory.

A total of 40 rectangular bar-shaped specimens were fabricated and randomly allocated into two experimental groups of 20 specimens each:

[1] Group A: Yttria-stabilized zirconia (Aizir, Aidite Technology, China)

[2] Group B: Lithium disilicate glass-ceramic (IPS e.max Press, Ivoclar Vivadent, Liechtenstein)

Sample size was determined based on previous studies evaluating fracture toughness of dental ceramics, with power analysis indicating that 20 specimens per group would provide 80% power to detect a clinically meaningful difference of 1.5 MPa.m^1/2^ at α = 0.05 significance level.

## Specimen preparation:

All specimens were fabricated with standardized dimensions of 17 mm length x 4 mm width x 1 mm thickness, conforming to ISO 6872:2015 specifications for testing dental ceramics.

## Zirconia specimen fabrication:

Pre-sintered zirconia blocks were scanned using an extraoral laboratory scanner (Rainbow Scanner, Dentium, South Korea). Digital design of rectangular specimens was performed using CAD software and specimens were milled from pre-sintered zirconia blanks using a 5-axis milling machine (Rainbow Mill, Dentium, South Korea), accounting for 25% sintering shrinkage. Milled specimens were cleaned ultrasonically in distilled water for 10 minutes and allowed to dry completely. Sintering was performed in a high-temperature furnace (Programat S1, Ivoclar Vivadent) following the manufacturer's protocol: heating rate of 10°C/min to 900°C, followed by 3°C/min to final sintering temperature of 1530°C, held for 2 hours, then cooled to room temperature at controlled rate of 5°C/min. Post-sintering dimensions were verified using digital calipers (accuracy ±0.01 mm).

## Lithium disilicate specimen fabrication:

For lithium disilicate specimens, wax patterns of identical dimensions were milled from wax blanks using the same CAD/CAM system. Wax patterns were invested using phosphate-bonded investment material (IPS PressVEST Premium, Ivoclar Vivadent) mixed under vacuum at high speed for 60 seconds to eliminate air bubbles. After complete setting (45 minutes), investment rings were placed in a burnout furnace with controlled heating protocol: 2°C/min to 250°C (held 30 min), 3°C/min to 400°C (held 30 min), then 5°C/min to final burnout temperature of 850°C (held 60 min). Hot-pressing was performed using a hydraulic press furnace (Programat EP 5010, Ivoclar Vivadent) at 920°C with controlled pressure of 0.3 MPa for 20 minutes. After cooling to room temperature, investment material was removed by airborne particle abrasion using 50 µm aluminum oxide at 2 bar pressure. Pressed specimens were finished using fine-grit diamond rotary instruments under water cooling and then ultrasonically cleaned in distilled water for 15 minutes.

## Surface preparation:

All specimens underwent standardized surface finishing ensuring uniform surface conditions for testing. Specimens were sequentially polished using silicon carbide abrasive papers of progressively finer grits (600, 800, 1000 and 1200 grit) under water lubrication. Each polishing step was performed for 2 minutes with consistent hand pressure. Final polishing was performed using 1 µm diamond paste on felt wheels for 3 minutes. Specimens were ultrasonically cleaned in distilled water for 10 minutes between each polishing step and finally cleaned in 96% ethanol for 5 minutes and air-dried. Surface roughness was verified using a profilometer, ensuring Ra values below 0.2 µm for all specimens. Specimens were stored in distilled water at 37°C for 24 hours before testing to simulate oral conditions.

## Vickers indentation testing:

Fracture toughness was determined using the Vickers indentation fracture method. Testing was performed using a digital micro-hardness tester (HVS-1000, Huatec, China) equipped with a Vickers diamond pyramid indenter (square-based pyramid with face angle of 136°).

## Hardness determination:

Initial indentations were made at 500 gf (4.9 N) load for 15 seconds to determine Vickers hardness. Five indentations were made on each specimen with minimum spacing of 1 mm between indentations and 0.5 mm from specimen edges. Diagonal lengths of indentations were measured using the integrated optical microscope at 400x magnification and Vickers hardness number (HV) was calculated automatically by the software.

## Fracture toughness testing:

For fracture toughness evaluation, indentation load was increased to 1000 gf (9.81 N) with 15-second dwell time. This load was selected based on pilot studies to ensure development of well-defined radial cracks extending from indentation corners. Three indentations were made on each specimen following the same spacing protocol. Immediately after indentation, specimens were examined using an optical microscope at 100x magnification and crack lengths were measured from the center of indentation to crack tips along both diagonals. Measurements were performed by a single calibrated examiner using image analysis software (ImageJ, NIH, USA) calibrated against a stage micrometer.

Where:

[1] K_IC_ = fracture toughness (MPa.m^1/2^)

[2] E = elastic modulus (210 GPa for zirconia, 95 GPa for lithium disilicate)

[3] H = Vickers hardness (GPa)

[4] P = indentation load (N)

[5] C = crack length measured from indentation center (m)

## Statistical analysis:

Data were recorded in Microsoft Excel 2019 and analyzed using SPSS Statistics version 25.0 (IBM Corp., USA). Descriptive statistics including mean, standard deviation, minimum and maximum values were calculated for all parameters. Normality of distribution was assessed using Shapiro-Wilk test. Since data followed normal distribution, independent samples t-test was employed to compare fracture toughness and hardness values between groups. Statistical significance was set at p <0.05. Intra-examiner reliability for crack length measurements was assessed using intraclass correlation coefficient (ICC) on 20% of randomly selected specimens measured twice with one-week interval.

## Results:

All 40 specimens were successfully fabricated and tested without any failures during preparation or testing procedures. Intra-examiner reliability for crack length measurements showed excellent agreement (ICC = 0.96, 95% CI: 0.92-0.98). Vickers hardness values demonstrated significant differences between the two ceramic materials. Zirconia specimens exhibited substantially higher hardness compared to lithium disilicate specimens. The mean Vickers hardness of zirconia (1322.35 ± 54.89 HV) was significantly higher than lithium disilicate (767.10 ± 46.25 HV), representing approximately 72.4% greater hardness (p <0.0001). Crack lengths measured from the indentation center showed inverse relationship with material resistance to crack propagation. Lithium disilicate specimens demonstrated longer crack propagation compared to zirconia under identical loading conditions (Table 1 - see PDF). Zirconia specimens exhibited significantly shorter crack lengths (0.106 ± 0.014 mm) compared to lithium disilicate (0.129 ± 0.009 mm), indicating superior resistance to crack propagation (p <0.0001). The coefficient of variation was higher for zirconia (13.21%) compared to lithium disilicate (6.98%), suggesting more variability in crack resistance behavior. The primary outcome measure, fracture toughness, revealed significant differences between the two ceramic systems, with zirconia demonstrating superior performance (Table 2 - see PDF). Zirconia demonstrated mean fracture toughness of 408.61 ± 44.21 MPa.m^1/2^, which was significantly higher than lithium disilicate (359.40 ± 19.00 MPa.m^1/2^), representing a 13.7% improvement (p <0.0001). The large effect size (Cohen's d = 1.446) indicates a substantial practical difference between materials. The null hypothesis was rejected, confirming significant differences in fracture toughness between zirconia and lithium disilicate ceramics. Individual specimen analysis revealed that all zirconia specimens accept two demonstrated fracture toughness values exceeding 380 MPa.m^1/2^, while 85% of lithium disilicate specimens showed values below 380 MPa.m^1/2^. The range of fracture toughness values was broader for zirconia (155 MPa.m^1/2^) compared to lithium disilicate (69.3 MPa.m^1/2^), indicating greater variability in the zirconia group (Table 3 - see PDF).

## Discussion:

The present investigation compared the fracture toughness of two widely used all-ceramic systems-yttria-stabilized zirconia and lithium disilicate glass-ceramic-using the Vickers indentation method. The findings revealed that zirconia exhibited significantly superior fracture toughness compared to lithium disilicate, thereby rejecting the null hypothesis. These results have important clinical implications for material selection in restorative dentistry, particularly for prostheses subjected to high occlusal stresses. The significantly higher fracture toughness of zirconia (408.61 ± 44.21 MPa.m^1/2^) compared to lithium disilicate (359.40 ± 19.00 MPa.m^1/2^) can be attributed to fundamental differences in microstructure and toughening mechanisms. Zirconia's exceptional mechanical properties arise primarily from transformation toughening; a unique phenomenon wherein stress-induced phase transformation from metastable tetragonal to monoclinic structure creates volumetric expansion (approximately 3-5%) that generates compressive stresses around crack tips, effectively shielding them from propagating forces [21]. This stress-induced transformation toughening mechanism represents the most significant strengthening feature of Y-TZP ceramics and distinguishes them from other ceramic systems [22]. Recent investigations using advanced characterization techniques have demonstrated that the transformation zone ahead of crack tips in zirconia can extend several micrometers, creating a process zone that absorbs substantial fracture energy [23]. Furthermore, the polycrystalline structure of zirconia with minimal glassy phase contributes to crack deflection at grain boundaries, adding another dimension to its toughening mechanisms [24]. Studies by Elsayed *et al.* have shown that the combination of transformation toughening and crack deflection can account for fracture toughness improvements of 300-400% compared to non-transforming ceramics [25]. The fracture toughness values obtained for zirconia in the present study align with reported ranges in the literature, though some variations exist. Zhang and Lawn reported fracture toughness values of 4-6 MPa.m^1/2^ for dental Y-TZP using various testing methods [26]. The relatively higher values observed in this study may be attributed to differences in calculation methodology inherent to the Vickers indentation technique, specimen preparation protocols and specific material composition. It is well-established that fracture toughness values obtained through indentation methods tend to yield higher absolute values compared to standardized fracture mechanics approaches such as single-edge V-notch beam or chevron-notch methods [27]. For lithium disilicate, the observed fracture toughness values (359.40 ± 19.00 MPa.m^1/2^) demonstrate the material's respectable mechanical performance, which can be explained by its unique microstructural characteristics. Lithium disilicate glass-ceramics contain approximately 70 vol% needle-like lithium disilicate crystals (ranging from 0.5 to 5 µm in length) embedded in a residual glassy matrix [28]. These elongated crystals create a complex, interlocking microstructure that promotes crack deflection, crack bridging and crack branching-all extrinsic toughening mechanisms that increase the energy required for crack propagation [29]. Recent research has highlighted the importance of crystal size distribution in lithium disilicate ceramics. Huang *et al.* demonstrated that materials with bimodal crystal size distribution-featuring both large rod-like crystals and smaller equiaxed crystals-exhibit superior fracture toughness compared to those with uniform crystal size [30]. The hot-pressing fabrication technique used in this study for IPS e.max Press promotes such bimodal distribution, contributing to optimal mechanical performance. However, the absence of phase transformation toughening limits lithium disilicate's fracture resistance compared to zirconia [31]. The significantly higher Vickers hardness observed for zirconia (1322.35 ± 54.89 HV) compared to lithium disilicate (767.10 ± 46.25 HV) correlates with the materials' crystal structures and phase compositions. Zirconia's fully crystalline structure with strong ionic-covalent bonding and minimal glassy phase contributes to exceptional hardness [32]. In contrast, lithium disilicate contains approximately 30% residual glassy matrix, which, while beneficial for esthetics and machinability, reduces overall hardness [33]. This hardness differential has clinical implications: while higher hardness generally correlates with wear resistance, excessive hardness may lead to accelerated wear of opposing dentition, a consideration in material selection [34]. The inverse relationship between crack length and fracture toughness observed in this study validates the fundamental principles of fracture mechanics. Zirconia specimens exhibited shorter crack lengths (0.106 ± 0.014 mm) compared to lithium disilicate (0.129 ± 0.009 mm) under identical indentation loads, reflecting superior crack growth resistance. This phenomenon can be quantitatively understood through the fracture toughness equation, where shorter crack lengths at equivalent loads directly translate to higher K_IC_ values [35]. The greater variability in zirconia's fracture toughness values (coefficient of variation: 10.82%) compared to lithium disilicate (5.29%) merits discussion. This increased variability may reflect the sensitivity of transformation toughening to subtle variations in microstructural features such as grain size, yttria distribution and residual stresses from sintering [36]. In contrast, the hot-pressing fabrication method for lithium disilicate may produce more consistent microstructures, leading to more uniform mechanical properties. Clinical implications of this variability suggest that while zirconia offers superior mean fracture toughness, quality control during manufacturing is critical to ensure consistent performance [37]. From a clinical perspective, the superior fracture toughness of zirconia supports its use in high-stress applications including multi-unit posterior fixed dental prostheses, implant abutments and full-arch rehabilitations [38]. The material's exceptional crack propagation resistance reduces the risk of catastrophic failure, potentially improving long-term survival rates. Conversely, lithium disilicate, despite lower fracture toughness, offers advantages in esthetically demanding situations due to superior translucency and more conservative preparation requirements [39]. Recent clinical studies have reported excellent 5-year survival rates (>95%) for lithium disilicate single crowns in both anterior and posterior regions, suggesting adequate mechanical performance for these applications [40]. The choice between these materials should be guided by evidence-based risk assessment considering multiple factors: restoration type, location, occlusal forces, preparation design and esthetic requirements. For high-risk scenarios-extensive posterior bridges, patients with parafunctional habits, or reduced connector dimensions-zirconia's superior fracture toughness provides a safety margin that may justify compromising on esthetics [41]. For single-unit anterior restorations or cases demanding optimal esthetics, lithium disilicate's adequate fracture toughness combined with superior optical properties may be preferable [42]. Recent advances in ceramic technology have aimed at combining the advantages of both materials. Multilayer zirconia with gradient translucency offers improved esthetics while maintaining mechanical strength [43]. Similarly, lithium silicate ceramics (containing both lithium disilicate and lithium metasilicate phases) have been developed to enhance machinability while preserving mechanical properties [44]. Future research should focus on comparing these newer material generations and investigating how surface treatments, cementation protocols and aging conditions affect long-term fracture resistance. Several limitations of this study warrant acknowledgment. First, the Vickers indentation method, while convenient and widely used, has been criticized for potential overestimation of absolute fracture toughness values compared to standardized fracture mechanics tests [45]. The technique's sensitivity to factors such as indentation load, residual stresses and slow crack growth may introduce variability. However, for comparative purposes between materials tested under identical conditions, the method provides reliable ranking [46]. Second, this study evaluated fracture toughness under static loading conditions at room temperature, which may not fully replicate the dynamic, cyclic loading and thermally variable oral environment. Fatigue testing under simulated oral conditions would provide additional clinically relevant data [47]. Third, the study examined specific commercial brands; results may not be generalizable to all zirconia or lithium disilicate products, as composition and processing variations among manufacturers can influence mechanical properties [48]. Despite these limitations, the study's strengths include standardized specimen preparation, adequate sample size with statistical power, rigorous surface finishing protocols and blinded measurements by a calibrated examiner with excellent reliability. The findings contribute valuable comparative data to guide evidence-based material selection in clinical practice. Future investigations should incorporate aging protocols simulating long-term oral conditions, including thermocycling, pH cycling and mechanical fatigue testing. Additionally, fractographic analysis using scanning electron microscopy could provide insights into failure modes and crack propagation patterns, enhancing understanding of material behavior under stress [49]. Three-dimensional finite element analysis correlating mechanical properties with stress distribution in various prosthetic designs would facilitate translation of laboratory data to clinical applications [50].

## Conclusion:

Zirconia consistently demonstrated superior mechanical performance, showing higher fracture toughness, hardness and resistance to crack propagation than lithium disilicate. These properties make zirconia more suitable for high-stress posterior and multi-unit restorations, while lithium disilicate remains ideal where esthetics outweigh functional load. Overall, material selection should balance biomechanical demands and esthetic needs to ensure long-term clinical success.
